# The Potential Effects of Dietary Antioxidants in Obesity: A Comprehensive Review of the Literature

**DOI:** 10.3390/healthcare12040416

**Published:** 2024-02-06

**Authors:** Noha M. Almoraie, Israa M. Shatwan

**Affiliations:** Food and Nutrition Department, Faculty of Human Sciences and Design, King Abdulaziz University, Building 43, Room 233, Level 2, Jeddah 3270, Saudi Arabia; eshatwan@kau.edu.sa

**Keywords:** dietary antioxidants, antioxidant enzymes, obesity, adiposity, oxidative stress, dietary supplementation

## Abstract

Obesity has become a global health concern, with its prevalence steadily increasing in recent decades. It is associated with numerous health complications, including cardiovascular diseases, diabetes, and certain types of cancer. The aetiology of obesity is multifactorial, involving genetic, environmental, and lifestyle factors. In recent years, oxidative stress has emerged as a potential contributor to obesity and its related metabolic disorders. Dietary antioxidants, which can counteract oxidative stress, have gained significant attention for their potential role in preventing and managing obesity. This comprehensive review aims to explore the impact of dietary antioxidants on obesity and its associated metabolic dysregulations, discussing the underlying mechanisms and highlighting the potential therapeutic implications.

## 1. Introduction

In the contemporary world, characterised by sedentary lifestyles and the prevalence of processed foods, the global obesity rates have reached unprecedented levels. Obesity a health condition marked by the excessive buildup of body fat and can adversely affect individuals’ well-being [1,2]. According to the World Health Organization, obesity is identified as having a body mass index (BMI) of 30 or higher [3]. The prevalence of this condition has escalated to widespread proportions over the past 50 years [4]. Recent studies indicate that over 670 million adults globally were classified as having obesity, alongside 1.3 billion deemed overweight, in 2016 [4,5].

The prevalence of obesity has significantly increased in North America [5], Western Europe [6], and the Gulf region [7]. According to a recently published systematic review of pooled data from 2000 to 2020, the prevalence of obesity and overweight in individuals in the Middle East was 21% and 33%, respectively [8].

Obesity is a complex health issue that can be influenced by diverse factors, including genetics, lifestyle, and dietary choices [9,10]. In the search for effective strategies to prevent and manage obesity, the role of dietary antioxidants has gained significant attention. Dietary antioxidants are natural compounds found in several foods, including fruits, vegetables, nuts, and whole grains [11]. They are crucial in protecting the body against oxidative stress, which results from an imbalance between generated harmful free radicals and the body’s capacity to neutralise them [12]. Oxidative stress has been implicated in the development and progression of obesity because it can lead to chronic inflammation and the dysfunction of metabolic processes [12,13]. Numerous studies propose that dietary antioxidants may have a potential impact on the prevention and control of obesity. These compounds have been found to possess anti-inflammatory properties and can help to regulate cellular processes involved in energy metabolism and adipose tissue function. Additionally, they may assist in reducing oxidative stress and improving insulin sensitivity, which are key factors in maintaining a healthy body weight [12,13].

The specific antioxidants that have shown promise in relation to obesity include vitamins C [12,14,15] and E, beta-carotene, selenium [15], and polyphenols [16]. These compounds have been studied for their potential effects on body composition, metabolic rate, fat oxidation, and appetite regulation [12,14,15,16,17]. However, research in this field is still emerging, and additional research is required to thoroughly understand the underlying mechanisms and optimal dosages of dietary antioxidants for obesity prevention and treatment. This comprehensive review aims to delve into the impact of dietary antioxidants on obesity, providing an extensive examination of their potential benefits and elucidating the underlying mechanisms involved.

## 2. Methodology of Search

Both primary and secondary sources were encompassed in this comprehensive literature review. Various scientific articles, bibliographic indexes, and databases were utilised to gather the relevant literature. The sources included Scopus, PubMed, Science Direct, Embase, ResearchGate, Sports Discuss, and Web of Science. The search was carried out utilising keywords compliant with Medical Subject Headings (MeSH) to guarantee the relevance of the collected literature. Specifically, keywords such as dietary antioxidants, obesity, antioxidant therapy, oxidative stress, adiposity, body mass index, inflammation, metabolic syndrome, energy metabolism, insulin sensitivity, lipid peroxidation, free radicals, antioxidant enzymes, nutritional interventions, and dietary supplementation were employed. The authors considered studies that met the following criteria for inclusion: (1) focused on the effects of dietary antioxidants in relation to obesity; (2) examined the impact of dietary antioxidants on relevant outcomes related to obesity, such as body weight, BMI, adiposity, inflammation, metabolic syndrome, insulin sensitivity, lipid peroxidation, and oxidative stress; (3) involved human participants or animal models of obesity; (4) were published between 2000 and November 2023; (5) were written in the English language. This approach aimed to retrieve studies that addressed the specific objectives of the review. To ascertain the suitability of the studies incorporated in the analysis, the authors thoroughly scrutinised the titles and abstracts of all retrieved manuscripts. Exclusion criteria were implemented, including eliminating studies that utilised outdated data beyond the specified timeframe, those unrelated to the specific objectives of this research, and those presented in other languages besides English. Upon identifying the pertinent studies, the reviewers autonomously retrieved information from the selected articles. This process was pivotal in upholding the quality and dependability of the data incorporated in the review. Cooperative discussions were undertaken among the reviewers to amalgamate the findings and present a narrative review. Through the amalgamation of the findings based on their expertise and perspectives, the authors guaranteed a thorough literature analysis. Through this method, we sought to deliver a unified and informative narrative addressing the study’s objectives.

## 3. Mechanisms of Obesity Development

### 3.1. Overview of the Multifactorial Nature of Obesity

Obesity is a complex and multifactorial condition influenced by an interplay of genetic, environmental, behavioural, and metabolic factors [18]. Comprehending the diverse contributors to obesity is vital to devise effective prevention and treatment strategies. Its aetiology is not confined to a single cause; rather, it emerges from the interactions of various elements. Appreciating the multifactorial nature of obesity is key to formulating effective prevention and management strategies [19]. Genetics substantially contribute to obesity susceptibility. Specific genes can predispose individuals to weight gain and fat accumulation by influencing their metabolism, appetite regulation, and fat storage [20]. Nonetheless, the sole consideration of genetics fails to explain the escalating global obesity rates, underscoring the impact of environmental and lifestyle factors [18,20].

Environmental factors encompass a wide range of elements, including the built environment, access to healthy food options, socioeconomic status, cultural norms, and advertising [21]. The obesogenic environment, characterised by an abundance of high-calorie, processed foods and sedentary lifestyles, fosters weight gain. A lack of physical activity, increased screen time, and sedentary occupations significantly contribute to an energy imbalance and subsequent weight gain [22,23]. Behavioural elements also have a crucial impact on the development of obesity. For instance, unhealthy eating habits, such as the consumption of energy-dense foods, frequent intake of sugary drinks, and oversized portion sizes, lead to excessive caloric intake [18,24]. Moreover, emotional eating, responding to stress by eating, and irregular meal patterns are influential factors in weight gain [25]. The lack of physical activity, including diminished participation in regular exercise or engagement in sedentary behaviours, further compounds this issue [26]. Furthermore, socio-cultural factors influence individuals’ attitudes, beliefs, and behaviours concerning food and physical activity. Cultural norms, societal pressures, and social influences can significantly affect dietary choices and physical activity levels [26]. Specifically, cultural celebrations centred around food and the prevalence of unhealthy food options at social gatherings can lead to excessive calorie intake [26,27,28].

### 3.2. Oxidative Stress and Obesity

Oxidative stress is a physiological imbalance between the generation of reactive oxygen species (ROS) and the capacity of the body’s antioxidant defence mechanisms to neutralise and repair the resulting damage [29]. It plays a crucial role in various physiological processes and is implicated in numerous health conditions. ROS are highly reactive molecules that are generated naturally by products of cellular metabolism [30]. They can be produced through processes such as mitochondrial respiration, enzymatic reactions (e.g., NADPH oxidases), and exposure to external sources such as environmental pollutants, radiation, and certain drugs [31]. Oxidative stress occurs when the generation of ROS exceeds the capabilities of the body’s antioxidant defences [32,33]. This stress can detrimentally affect cellular components, such as lipids, proteins, and DNA. ROS are known to initiate lipid peroxidation, a chain reaction that damages cellular membranes [30]. Consequently, this leads to the formation of lipid peroxides and other reactive lipid entities, resulting in membrane disruption, altered membrane fluidity, and compromised cellular function [30]. Additionally, ROS can directly modify amino acid residues in proteins, causing protein oxidation. This oxidation may cause protein misfolding, altered enzymatic activity, and disrupted cellular signalling pathways [30,34]. The oxidation of proteins can also lead to the formation of protein aggregates and contribute to the onset of neurodegenerative diseases. Furthermore, ROS have the potential to cause oxidative harm to DNA, including alterations to the bases, breaks in DNA strands, and the formation of links between DNA and proteins. If such DNA damage remains unrepaired, it can result in mutations, genomic instability, and heightened vulnerability to various diseases, including cancer [34]. Oxidative stress has implications for various physiological processes in the body. It contributes to the aging process by causing cellular senescence, telomere shortening, and impaired mitochondrial function [30]. Excessive oxidative stress can trigger and perpetuate inflammation through the activation of redox-sensitive signalling pathways [30,35]. Moreover, inflammatory cells, such as macrophages, produce ROS as part of the immune response [35]. However, excessive ROS production can result in chronic inflammation, which is implicated in various chronic diseases. In terms of cardiovascular health, oxidative stress promotes endothelial dysfunction, lipid oxidation, vascular inflammation, and the formation of atherosclerotic plaques [36]. Neurodegenerative conditions such as Alzheimer’s and Parkinson’s diseases are linked to oxidative-stress-induced neuronal damage, protein aggregation, mitochondrial dysfunction, and neuroinflammation. Moreover, oxidative stress contributes to cancer development and progression through DNA damage, genetic mutations, and alterations in cellular signalling pathways [35]. Importantly, it is involved in the development of metabolic conditions such as obesity, type 2 diabetes, and non-alcoholic fatty liver disease, which result in insulin resistance and metabolic abnormalities [37].

### 3.3. Mechanisms Linking Oxidative Stress to Obesity: Impact on Adipocyte Dysfunction, Insulin Resistance, Chronic Inflammation, and Dysregulated Lipid Metabolism

Oxidative stress in obesity detrimentally affects various aspects of metabolic health. It disrupts the normal function of adipocytes, the cells responsible for storing and releasing fat [38]. The excessive production of ROS in adipocytes leads to adipocyte dysfunction, characterised by the altered secretion of adipokines (hormones produced by adipose tissue) and changes in adipocyte size and number [39]. This dysfunction can result in an impaired fat storage capacity and increased ectopic lipid deposition in non-adipose tissues such as the liver and muscle, contributing to metabolic disturbances [39,40]. Moreover, oxidative stress is important in the development of insulin resistance, a hallmark of obesity and type 2 diabetes [39]. Elevated levels of ROS interfere with insulin signalling pathways, leading to reduced insulin sensitivity in target tissues such as the adipose tissue, liver, and skeletal muscle [40,41]. ROS can directly impair insulin signalling by modifying insulin receptor substrates and downstream signalling molecules, hindering glucose uptake and utilisation. This disruption in insulin signalling promotes hyperglycaemia and compensatory hyperinsulinemia, ultimately contributing to the development of insulin resistance [42,43]. Moreover, the development of insulin resistance is influenced by the metabolism of lipids and the storage of excess fat calories in the adipose tissue, which promote increased free fatty acids in circulation. Free fatty acids stimulate the production of large amounts of free radicals in the body, which can damage cellular proteins [44]. Additionally, obesity is associated with chronic low-grade inflammation, which is defined as the initiation of the process at cellular levels and associated with increased plasmatic (e.g., C-reactive protein) or cellular biomarkers (e.g., white blood cell and platelet counts) [45,46], and oxidative stress is a key driver of this inflammatory response [12,35,36]. Adipose tissue, particularly visceral adipose tissue, is a significant source of ROS production in obesity [47]. Excessive ROS in adipose tissue triggers the activation of pro-inflammatory signalling pathways, such as nuclear factor kappa-light-chain-enhancer of activated B cells (NF-κB), leading to the secretion of pro-inflammatory cytokines (e.g., tumour necrosis factor-α, interleukin-6) and chemokines [48]. These inflammatory mediators further exacerbate the adipose tissue inflammation and contribute to systemic inflammation, which is associated with the onset of various metabolic disorders [48]. Inflammation represents a triggering factor in the development of metabolic syndrome, which is associated with a pro-inflammatory state. Low-grade inflammation, considered a diagnostic parameter associated with metabolic syndrome, is thought to be the central mechanism in the outcome pathophysiology. Overnutrition, physical inactivity, and ageing, which are causes of cytokine hypersecretion in chronic inflammation, lead to metabolic disorders in predisposed individuals [49,50]. Notably, oxidative stress, a key factor in obesity, disrupts lipid metabolism. This disruption can lead to dyslipidaemia, marked by abnormal lipid levels in the blood, alongside altered lipid storage and heightened lipotoxicity [51]. ROS-induced lipid peroxidation produces deleterious lipid metabolites, such as oxidised low-density lipoproteins and lipid peroxides. These metabolites can initiate inflammation, accelerate atherosclerosis, and compromise endothelial function [16,48,51]. Moreover, oxidative stress impairs the normal activity of peroxisome proliferator-activated receptors (PPARs), a critical group of transcription factors in lipid metabolism. The dysregulation of PPAR signalling results in imbalances in lipid storage, lipolysis, and fatty acid oxidation, thereby exacerbating the metabolic disturbances seen in obesity [52].

### 3.4. Role of Antioxidants in Combating Oxidative Stress: Modulating Pathophysiological Processes in Obesity

Antioxidants are pivotal in countering oxidative stress by neutralising ROS and safeguarding against oxidative harm. Functioning as scavengers, they donate electrons to ROS, thereby inhibiting cellular damage [53]. Particularly in obesity, oxidative stress significantly contributes to metabolic irregularities. Antioxidants, including vitamins C and E, beta-carotene, and a range of phytochemicals, actively neutralise ROS, diminishing their detrimental impact [17,54]. These agents provide electrons to ROS, stabilising them and preventing their interaction with cellular elements such as lipids, proteins, and DNA [30]. By neutralising ROS, antioxidants preserve the redox equilibrium and shield cells from oxidative injury. This aspect is especially crucial in obesity, where augmented ROS generation leads to adipocyte dysfunction, insulin resistance, chronic inflammation, and disrupted lipid metabolism [17]. Furthermore, antioxidants play a crucial role in regenerating endogenous antioxidant systems within cells [32]. Additionally, antioxidants like glutathione and NADPH are essential in maintaining the activity of key antioxidant enzymes, including superoxide dismutase, catalase, and glutathione peroxidase. Together, these enzymes neutralise ROS and maintain the cellular redox balance [31,55]. Vitamin C acts as a cofactor for fifteen enzymes and functions as an antioxidant by donating electrons. It plays a crucial role in scavenging free radicals and protecting tissues against oxidative stress, thereby reducing inflammation [56].

Oxidative stress and inflammation are closely interconnected in obesity. Antioxidants can modulate inflammatory signalling pathways by inhibiting the activation of transcription factors like NF-κB and activator protein-1 [57,58]. To suppress these pro-inflammatory pathways, antioxidants can reduce the production of pro-inflammatory cytokines and chemokines, thus mitigating the chronic inflammation associated with obesity [58]. Importantly, antioxidants have the potential to improve insulin sensitivity, a crucial factor in obesity-related insulin resistance. By reducing oxidative stress, antioxidants can help to restore normal insulin signalling pathways [42,43]. Notably, antioxidants such as alpha-lipoic acid and resveratrol have been demonstrated to enhance insulin sensitivity by activating the signalling pathways involved in glucose uptake and utilisation in skeletal muscle and adipose tissue [59,60]. Furthermore, antioxidants can modulate lipid metabolism in obesity. Specifically, a recent study revealed that polyphenols, found in fruits, vegetables, and tea, are able to inhibit lipogenesis and promote lipolysis. Through regulating lipid metabolism, antioxidants can help to prevent excessive lipid accumulation in the adipose tissue and ectopic lipid deposition in non-adipose tissues, thereby reducing the metabolic disturbances associated with obesity [61]. Excess fat accumulation can have harmful effects on organs, including hepatic steatosis, fibrosis of the liver, impaired insulin secretion, β-cell dysfunction, cardiomyopathy, and coronary heart disease [62].

## 4. Dietary Antioxidants and Obesity

### 4.1. Antioxidant-Rich Foods and Their Constituents: An Overview of Their Antioxidant Components

Antioxidants are commonly classified as natural or synthetic. Natural antioxidants, encompassing polyphenols, carotenoids, and vitamins, originate from food and plant sources [63,64]. Moreover, they are found in diverse plant components, including fruits, vegetables, nuts, seeds, leaves, roots, and barks [65]. They demonstrate a broad range of biological activity and have substantial nutritional value, predominantly owing to their antioxidative mechanisms. Moreover, these naturally occurring antioxidants are deemed safe for consumption and are not associated with any known side effects.

### 4.2. Insights into Modulation of Adipogenesis and Lipid Metabolism

Dietary antioxidants have emerged as potential modulators of these processes, with evidence suggesting their ability to influence adipocyte development and lipid handling. Several studies have investigated the effects of dietary antioxidants on body composition measures, including body weight, BMI, and fat mass. Some clinical trials have reported modest reductions in body weight and BMI following antioxidant interventions [18,19]. One study included 64 obese patients from primary healthcare centres in Gaza City, Palestine, who were hypertensive and/or diabetic and had high levels of inflammatory markers. These participants were enrolled in an open-label, parallel, randomised controlled trial, with 33 and 31 patients randomised into the control and experimental groups, respectively. The experimental group received a daily treatment of 500 mg vitamin C twice a day, while the control group did not receive any specific treatment. The results showed that the supplementation with vitamin C led to a significant decrease in body weight and BMI compared to the placebo group [66]. Individuals with obesity undergoing bariatric surgery were reported to have a vitamin C deficiency defined as any concentration ≤0.3 mg/dL [67,68]. Vitamin C levels are negatively correlated with BMI, waist-to-height ratio, and leptin concentrations [69]. A threshold impact analysis suggests that consuming more than 75 mg of vitamin C per day may be associated with lower obesity rates [70]. Importantly, vitamin C hinders the formation of mature fat cells, inhibits cell growth, blocks the breakdown of fats, regulates the release of glucocorticoids from the adrenal glands, and inhibits glucose metabolism and leptin secretion. These actions contribute to reduced hyperglycaemia and decreased glycosylation [71]. Despite these findings, the available data do not support the use of vitamin supplementation for obesity management [72]. Furthermore, research has shown a link between vitamin deficiencies and the accumulation of abdominal fat in individuals with obesity. For example, a review study revealed a negative relationship between vitamin C and total body fat [73]. Additionally, vitamin C has been found to prevent obesity by regulating lipid accumulation, inhibiting lipolysis, reducing glucocorticoid production, disrupting interactions between adipose cells and macrophages, scavenging ROS, and potentially inhibiting the hypoxia inducible factor-1a pathway [74].

While there is no association between vitamin E status and obesity markers [75,76], individuals with obesity with metabolic syndrome require more vitamin E due to the increased oxidative stress caused by their weight and other issues. However, these same problems result in the reduced utilisation of vitamin E [77]. In a case–control study conducted in Thailand, an inverse relationship was observed between BMI, waist and hip circumference, and the serum levels of vitamin E and retinol [78]. The impact of dietary antioxidants on metabolic markers linked to obesity has been extensively explored in numerous studies [75,76]. Clinical trials have reported enhancements in metabolic markers following antioxidant interventions, including a study that demonstrated that supplementation with vitamin E notably improved insulin sensitivity and decreased markers of oxidative stress in individuals with obesity and type 2 diabetes [76].

Numerous studies have begun exploring the relationship between antioxidant levels and fat accumulation in individuals with obesity. Aeberli et al. carried out a study on Swedish children, revealing a notable association between the consumption of antioxidant vitamins (like vitamin E, vitamin C, and β-carotene) and leptin levels [79]. This indicates that diminished levels of these vitamins might influence the genetic expression of leptin, potentially leading to leptin resistance and a heightened risk of obesity [79].

Elevated amounts of vitamin A might impede the maturation of adipocytes and indirectly impact insulin sensitivity by controlling the synthesis of bioactive proteins released by adipocytes, such as leptin and resistin [80]. Individuals with obesity were found to have reduced serum beta-carotene levels, possibly attributed to the predominant distribution of carotenoids in the serum and adipose tissue. Given that the adipose tissue serves as a significant storage site in humans, those with higher adiposity levels are likely to sequester a larger proportion of carotenoids in their adipose tissue compared to individuals with lower levels of adiposity [81,82]. This suggests that individuals with obesity may require higher intake of carotenoids to fulfil their antioxidant needs. Additionally, studies have shown that carotenoids have various benefits, such as improving insulin resistance, reducing adipocyte size and body fat tissue, lowering pro-inflammatory markers of obesity (e.g., low-density lipoprotein cholesterol (LDL-c) and very low-density lipoprotein cholesterol), and increasing high-density lipoprotein cholesterol (HDL-c), thus promoting prevention [83,84].

Selenium is renowned for its antioxidative characteristics and impact on the thyroid, immune system, and reproductive function. Several studies have confirmed a noteworthy connection between the intake of dietary selenium and obesity. For example, research indicated that individuals with higher selenium intake tended to gain weight, in contrast to those in the low-selenium group, who generally experienced weight loss [85]. Health conditions like liver cirrhosis and steatosis, frequently linked with obesity, have shown a positive association with heightened dietary selenium intake and increased blood selenium levels [86]. Furthermore, various studies have illustrated a link between dietary selenium intake, blood selenium concentrations, and an elevated risk of type 2 diabetes across diverse populations [87,88]. Excessive selenium exposure, as observed in animal experiments, has been linked to adverse outcomes, possibly due to the activation of insulin resistance mediated by selenoproteins. Recent evidence suggests that high selenium levels might lead to oxidative stress, impede biofilm development, and suppress enzyme activity [89]. Zinc, an essential nutrient, is crucial for growth, tissue repair, and immune function. Various studies have explored the relationship between zinc and obesity. Zinc deficiency has been associated with increased fat deposition and a higher body weight [90]. The interaction between zinc metabolism and leptin may shed light on the link between the zinc status and obesity. Zinc deficiency is known to decrease leptin levels in the blood, whereas zinc supplementation produces the opposite effect [91,92]. In experiments with mice, long-term zinc supplementation led to the accumulation of visceral adipose tissue without affecting the formation or breakdown of fat cells [93]. Mice are considered a good animal model because of their genetic similarities with humans and similar immune responses [94,95]. However, zinc alone does not function as a potent protective factor against obesity and only exerts a moderate impact in terms of averting weight gain. There are concerns that the consumption of zinc supplements might potentially compromise the immune system and reduce HDL cholesterol levels, possibly leading to copper deficiency [96]. The exact mechanism behind these effects still needs to be determined.

### 4.3. Dietary Antioxidants’ Influence on Energy Expenditure, Thermogenesis, Appetite, and Satiety

Recent studies have shed light on the influence of dietary antioxidants on energy expenditure, thermogenesis, appetite, and satiety, providing valuable insights into the comprehensive effects of antioxidants on metabolic homeostasis.

Several pieces of evidence suggest that antioxidants may have a significant impact on the regulation of hormones and neuropeptides related to appetite, satiety, energy expenditure, and maintaining balance in the body (such as leptin and adiponectin from adipose tissue; insulin from the pancreas; GLP-1, ghrelin, PYY, and CCK from the gastrointestinal system; and NPY, AgRP, and proopiomelanocortin (POMC)/CART at the hypothalamic level). This effect could potentially reduce the likelihood of obesity [97]. Clinical studies involving human participants support the utilisation of antioxidants in the context of obesity and highlight their advantageous impacts, particularly in terms of activating β-oxidation processes, enhancing satiety, boosting energy expenditure, suppressing adipocyte differentiation, facilitating adipocyte apoptosis, increasing lipolysis, and ameliorating disorders related to lipid metabolism [98].

Recent research efforts have focused on unravelling the molecular and genetic mechanisms underlying obesity and its associated conditions. A notable illustration of the regulation of food intake is the rise in food consumption, known as hyperphagia, following a period of fasting. The equilibrium between energy intake (food consumption) and energy expenditure (encompassing the basal metabolic rate, physical activity, and adaptive thermogenesis) is closely regulated. A homeostatic network manages the energy reserves by aligning the regulatory feeding centres in the central nervous system, particularly in the hypothalamus, with the controlled storage and release of fat stores that are essential to sustain the overall energy balance in the body. Therefore, genes encoding the molecular components of this system might play a role in the development of obesity and related disorders [99], including leptin, the leptin receptor, POMC, and the melanocortin 4 receptor [100]. Among the components of oolong tea, caffeine is recognised as a particularly potent anti-obesity agent [101]. Research has shown that caffeine can decrease food intake [102] and enhance thermogenesis, thereby contributing to weight loss [103]. Moreover, the thermogenic effect of caffeine was amplified when combined with catechins in the adipose tissue of rats [104]. A study indicated that higher dietary intake of antioxidants, specifically vitamins C and E, correlated with increased resting energy expenditure, suggesting a role for antioxidants in boosting energy expenditure at rest, which could aid in weight management [105]. Additionally, a comprehensive review of various studies revealed that certain antioxidants, such as capsaicin from chili peppers and catechins from green tea, may enhance thermogenesis and increase energy expenditure, implying that diets rich in these antioxidants could positively affect the metabolic rate and calorie burning [106]. Recent research has also underscored the potential of dietary antioxidants in modulating appetite and satiety. In a study examining the impact of polyphenol-rich blueberry supplementation on appetite and satiety, researchers observed heightened satiety and reduced subjective appetite in individuals with overweight and obesity, after consuming blueberries. The study involved 54 adults (27 in the blueberry group and 27 in the control group) with a BMI ≥ 25. After the first six weeks of treatment, individuals with overweight or obesity replaced 50 g of their carbohydrates with a 50 g serving of blueberries on a daily basis for a total of 12 weeks [107]. This study indicates that the polyphenols in blueberries might play a role in regulating appetite and enhancing feelings of fullness [107,108]. Collectively, these recent studies provide evidence supporting the influence of dietary antioxidants on energy expenditure, thermogenesis, appetite, and satiety. Nonetheless, it is crucial to acknowledge that these effects may differ based on variables such as the specific antioxidant compound, dosage, supplementation duration, and individual metabolic differences.

## 5. Challenges and Future Directions in Research

Investigating the effects of dietary antioxidants on obesity presents several challenges, including individual variability, lifestyle factors as confounders, and the complex, multifactorial nature of obesity [98,109]. Short-term studies may not adequately reflect the slow progression of obesity, and additional complications, such as adherence issues and the lack of uniform methodologies, can further skew research outcomes. To overcome these challenges, it is crucial for future studies to focus on long-term interventions to observe sustained impacts, engage in detailed mechanistic studies utilising advanced techniques, and investigate new sources and forms of antioxidants. These research approaches are essential in gaining a deeper insight into the interaction between dietary antioxidants and obesity, thereby contributing to the creation of effective, personalised prevention and treatment strategies [110,111].

The current understanding of the mechanisms and therapeutic potential of dietary antioxidants in obesity management reveals significant gaps, necessitating additional research. While existing studies offer valuable insights, the complex interplay of factors influencing the impact of antioxidants on obesity remains incompletely elucidated. The mechanistic pathways connecting antioxidant intake to physiological outcomes in obesity are intricate and require in-depth exploration. The optimisation of therapeutic strategies employing dietary antioxidants requires a more detailed understanding of variations in individual responses, dosage needs, and possible synergies with lifestyle interventions [112]. It is imperative to bridge these knowledge gaps through focused research, which is essential to fully realise the potential of dietary antioxidants in managing obesity. This will facilitate the creation of evidence-based, personalised approaches for enhanced therapeutic results.

Longitudinal studies and randomised controlled trials with extended intervention periods are essential to evaluate the prolonged impact of dietary antioxidants on obesity-related outcomes, including body weight, body composition, and metabolic parameters [13,113]. Furthermore, it is vital to examine how food processing might affect antioxidant content by researching prescribed concentrations of individual antioxidants or specific combinations that demonstrate a higher capacity to reduce oxidative stress in individuals with obesity. Moreover, exploring the potential advantages of novel antioxidants might offer fresh perspectives on their influence on obesity [114,115]. Thus, addressing these research gaps will facilitate a more comprehensive understanding of the mechanisms underlying the effects of dietary antioxidants on obesity.

## 6. Conclusions

Obesity is the one of the most prevalent metabolic disorders affecting public health. This review shows that oxidative stress not counteracted by an intact antioxidant defence system plays an important role in in various health conditions, including obesity. In addition, oxidative stress contributes to other metabolic conditions, such as insulin resistance and non-alcoholic fatty liver disease. The mechanisms linking oxidative stress to obesity involve detrimental effects on adipocyte function, insulin resistance, chronic inflammation, and dysregulated lipid metabolism. Nutrition can mediate the effect of oxidative stress by neutralising ROS and protecting against oxidative damage, and dietary antioxidants represent the best therapy. Studies on the impact of antioxidants on obesity were discussed. The findings suggested that dietary antioxidants, including vitamins C and E, beta-carotene, and various phytochemicals, had positive effects on body weight, BMI, fat mass, and metabolic markers. Despite the positive effects, the recommendation is to obtain antioxidants from a healthy, balanced diet rich in fruits, vegetables, and grains, and not from supplementation. However, challenges in research, including individual variability, confounding lifestyle factors, and the multifactorial nature of obesity, necessitate further investigation. Long-term studies, mechanistic exploration, and the examination of new antioxidant sources are crucial for a comprehensive understanding of the therapeutic potential of dietary antioxidants in obesity management. Addressing these challenges will contribute to the development of evidence-based and personalised approaches for effective obesity prevention and treatment.

## Data Availability

Not applicable.
